# A Recent Review of Electrospun Porous Carbon Nanofiber Mats for Energy Storage and Generation Applications

**DOI:** 10.3390/membranes13100830

**Published:** 2023-10-13

**Authors:** Al Mamun, Mohamed Kiari, Lilia Sabantina

**Affiliations:** 1 Junior Research Group “Nanomaterials”, Faculty of Engineering and Mathematics, Bielefeld University of Applied Sciences and Arts, 33619 Bielefeld, Germany; 2 Department of Physical Chemistry, Institute of Materials, University of Alicante, 03080 Alicante, Spain; 3 Faculty of Apparel Engineering and Textile Processing, Berlin University of Applied Sciences—HTW Berlin, Hochschule für Technik und Wirtschaft Berlin, 12459 Berlin, Germany

**Keywords:** electrospinning, porous carbon nanofibers, energy storage, nanofibers, dye-sensitized solar cells (DSSCs), biosensors

## Abstract

Electrospun porous carbon nanofiber mats have excellent properties, such as a large surface area, tunable porosity, and excellent electrical conductivity, and have attracted great attention in energy storage and power generation applications. Moreover, due to their exceptional properties, they can be used in dye-sensitized solar cells (DSSCs), membrane electrodes for fuel cells, catalytic applications such as oxygen reduction reactions (ORRs), hydrogen evolution reactions (HERs), and oxygen evolution reactions (OERs), and sensing applications such as biosensors, electrochemical sensors, and chemical sensors, providing a comprehensive insight into energy storage development and applications. This study focuses on the role of electrospun porous carbon nanofiber mats in improving energy storage and generation and contributes to a better understanding of the fabrication process of electrospun porous carbon nanofiber mats. In addition, a comprehensive review of various alternative preparation methods covering a wide range from natural polymers to synthetic carbon-rich materials is provided, along with insights into the current literature.

## 1. Introduction

The escalating energy demand, driven by global population growth and improved living standards, poses a paramount challenge. This strain on conventional fossil fuel reserves raises concerns about energy security and geopolitical tensions. Furthermore, fossil fuel combustion compounds environmental issues, emitting pollutants and greenhouse gases and contributing to climate change. Addressing these challenges hinges on research and innovation. Progress in renewable energy technologies, energy storage, smart grids, and energy-efficient materials can foster a more sustainable energy landscape. The development of efficient energy storage and generation technologies is of paramount importance in meeting our society’s growing energy needs while reducing environmental impact [1,2,3]. In this context, electrospun nanofibers play an important role as they offer promising solutions to these challenges due to their unique structure and chemical properties. Due to the exceptional physical and chemical properties of electrospun nanofibers, their potential in this field is considerable. These properties include an intricate network of nano- and microporous structures, a high surface-to-volume ratio, tunable porosity, and a variety of surface functionalities and electrical conductivities. These nanofibers can be fabricated from a wide range of polymers, including ceramics. In addition, these nanofiber mats can be electrospun from bio- or natural polymer solutions. After that, they can be easily converted into carbon nanofiber mats through stabilization and carbonization processes [4,5,6]. For example, in the study conducted by Jeon et al., they successfully produced porous carbon nanofibers with excellent energy storage capabilities using a single precursor polymer, polyimides containing hexafluoroisopropylidene diphthalic anhydride and 2-2′-bis(trifluoromethyl)benzidine (6FDA-TFMB), while bypassing the need for pore-forming substances. The process involved the synthesis of 6FDA-TFMB, followed by electrospinning and subsequent thermal treatment, resulting in binder-free carbon nanofiber electrodes for use in electrochemical double-layer capacitors [7]. The research of porous carbon nanofibers and membranes has attracted considerable attention from the scientific community in recent years. Numerous studies and publications have already highlighted the diverse applications of these materials. However, there are also controversial and differing hypotheses regarding their properties and areas of application. Despite this growing interest, however, there is a lack of comprehensive reviews that systematically summarize the various aspects and applications of these materials [8,9,10].

Therefore, the main objective of this review is to provide an up-to-date and comprehensive overview of the state of the art in the field of electrospun porous carbon nanofibers and membranes for energy storage and generation applications. Different production technologies, such as electrospinning, template-based synthesis, chemical vapor deposition (CVD), and activation processes, are considered in detail. In addition, this review will also discuss the diverse applications of porous carbon nanofibers in energy storage and power generation. These include their use in (DSSCs), membrane electrodes for fuel cells, catalytic applications such as oxygen reduction reactions (ORRs), hydrogen evolution reactions (HERs), and oxygen evolution reactions (OERs), and sensor applications such as biosensors, electrochemical and chemical sensors, to name but a few. Finally, the main findings of this review are summarized, and an outlook on future research directions is given.

## 2. Manufacturing Technologies and Surface Modification Processes

There are different types of methods for the synthesis of porous materials, such as chemical vapor deposition (CVD), template-based synthesis, electrospinning, etc. [11]. In particular, the electrospinning process is a new, simple, and environmentally friendly way to produce porous-structured materials. In the following, some fabrication methods for porous nanostructured carbon materials are discussed [12,13,14].

### 2.1. Electrospinning

**Figure 1:**
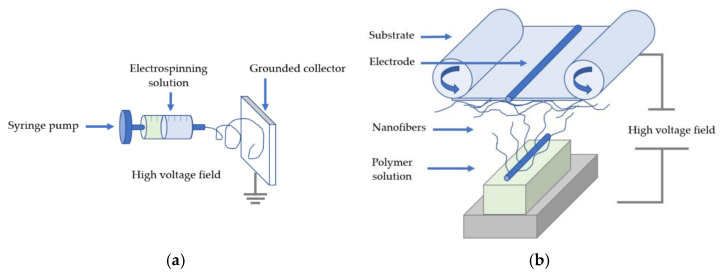
Basic set-up for (**a**) needle-based and (**b**) needle-less electrospinning technique. Reprinted from Ref. [28], originally published under a CC-BY license.

Interest in nanofiber mats has grown steadily, leading to an increase in research and publications [15]. Scientific literature has reported various methods for producing nanofibers, each method having its own approach. These methods include melt electrospinning, coaxial electrospinning, multi-nozzle electrospinning, needleless electrospinning, bubble electrospinning, electro-blowing, cylindrical porous hollow tube electrospinning, self-bundling electrospinning, and charge injection electrospinning [16,17]. Among all of the processes, electrospinning, including needle and needle-free, has become a very popular and widely used method for the production of nanofiber mats. The simplicity of the process, its compatibility with various polymers and admixtures, and the ability to produce nanofibers with tailored properties make it a preferred choice in the scientific community [18,19]. In addition, the electrospinning process is a relatively uncomplicated technological process that can produce continuous and extremely fine fibers or fiber mats with diameters in the nanometer range [20,21]. This technique can be used to produce ultrafine fibers from a variety of materials, including polymer composites or melts and inorganic or organic materials [22,23,24]. The materials used in this process can include various components such as ceramics, metallic nanoparticles, particles, carbon nanotubes, and other similar materials. It has gained popularity due to its relative simplicity and effectiveness in producing nanofibers with large surface area, fine diameters, and controllable morphology [25,26,27]. Basic needle-based electrospinning can be configured vertically or horizontally. For needle-based electrospinning, there are two variants: rotating and stationary spinnerets. Spinnerets connected to a high-voltage source and a solution dosing unit significantly affect the quality and productivity of nanofibers. Nanofiber production requires precise control and a reservoir for the polymer solution, usually a small-diameter syringe. Needleless electrospinning, on the other hand, is a self-assembled process that enables the direct fabrication of nanofibers from a liquid surface. In this process, a thin layer of a polymer solution is deposited on the spinneret and rotates, forming conical tips. At the same time, electrical forces create Taylor cones that lead to the formation of nanofibers. Decisive factors are the properties of the solution, the operating parameters, and the ambient conditions [28]. Figure 1 illustrates the basic configuration for both needle-based and needleless electrospinning processes.

**Figure 2:**
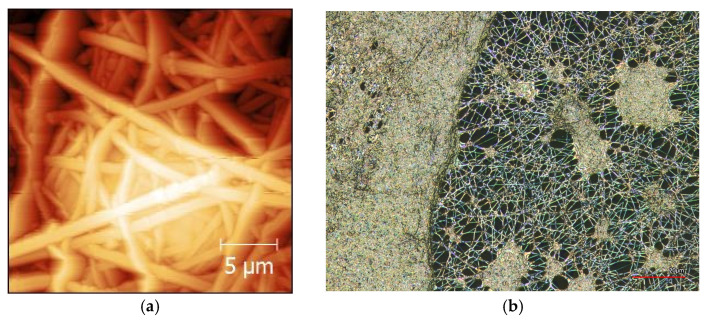
Atomic force microscope (AFM) (**a**) and confocal laser scanning microscope (CLSM) (**b**) images of magnetic electrospun PAN nanofiber mat with 50 wt% Fe_3_O_4_. Scale bars indicate 5 μm and 20 μm.

In this process, an electric field is applied to a polymer solution or melt, resulting in the formation of a charged jet that expands and solidifies into nanofibers as the solvent evaporates or cools. This technique enables the fabrication of nanofiber mats with desired properties such as high porosity, specific surface functionality, and tunable mechanical properties [29]. Figure 2 shows an atomic force microscope (AFM) and confocal laser scanning microscope (CLSM) images of magnetic electrospun PAN nanofiber mats with 50 wt% Fe_3_O_4_. 

The fabrication of carbon nanofibers by electrospinning involves the use of carbon-based precursors and electrospinning technology [30]. CNFs are nanostructured carbon materials with high aspect ratios and unique properties that make them desirable for various applications such as energy storage, catalysis, sensors, and reinforcement of composite materials [31]. The electrospinning process for producing carbon nanofibers typically begins with the preparation of a precursor solution. Zong et al. have provided an overview of the basic mechanisms of electrospinning and spraying techniques. They have summarized the reliable synthesis of nanomaterials, scaffolds, and membranes using these methods. They have also provided an overview of potential applications related to lithium batteries [32]. This solution includes a carbon source, which may be a polymer or a carbon-containing material dissolved in a suitable solvent. Common carbon precursors used in electrospinning include polyacrylonitrile (PAN), pitch, and various natural or synthetic polymers with a high carbon content. For CNFs, the heat treatment or carbonization step is essential to remove the non-carbon components and convert the precursor material into a carbonaceous structure [33].

Electrospinning is a versatile method for producing nanofibers, offering benefits like tailored material properties and controlled morphology. It finds applications in various fields. However, challenges include the need for precise parameter control, slow production rates, and safety precautions due to high voltage usage.

### 2.2. Template-Based Synthesis

**Figure 3:**
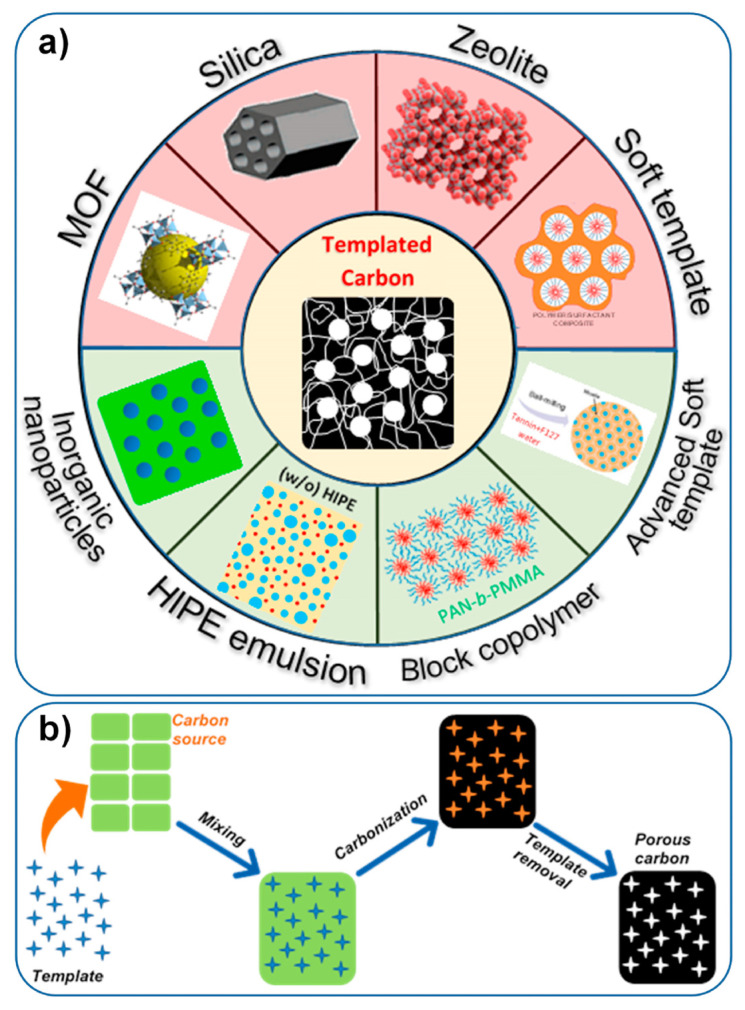
(**a**) Synthesis approaches towards templated porous carbons and (**b**) schematic illustration of the endo-templating method. Reprinted from Ref. [51], originally published under a CC-BY license.

One common method for preparing porous CNFs involves using templates, such as electrospun polymer fibers or sacrificial templates like silica or metal oxide particles. The carbon precursor is deposited onto the template, followed by subsequent carbonization and template removal steps. This approach allows for precise control over the size, shape, and distribution of the pores within the nanofibers. Over the past 40 years or so, a variety of template synthesis techniques have been shown to produce porous solids, especially ordered porous solids like porous carbons [34,35,36,37], ordered mesoporous silicas, and metal oxides [38,39,40,41], as well as ordered macroporous materials [42]. Recently, the different templates have been divided into endo-templates and exo-templates, two distinct groups. In the first type, a template is an independent object that can absorb into the developing solid and whose removal leaves the desired pore system in its place. The second is a porous framework that acts as a support system for a porous (or nonporous) solid. The family of porous carbons, which includes activated carbon, is significant [43,44,45,46]. The most typical process for making activated carbons involves carbonizing a precursor that contains carbon, followed by activation or post-treatment [47]. Techniques for controlling the pore size of activated carbons have been the focus of research for several decades due to the practical requirements of various applications. These techniques include high burn-off activation, catalyst-assisted activation, and carbonization of polymer blends with thermally unstable components. The production of carbons with predefined porous structural properties is difficult, necessitating the employment of specialized techniques, which raises the cost. Given their distinctive utility and adaptability, template techniques have been intensively researched among these strategies [48,49]. The idea behind template-assisted synthesis is that one or more precursors, or the results of their interaction, serve as a pattern or template that enables the synthesis of porous carbons. The geometric structure of the resultant porous material, in the simplest scenario, is an opposite copy of the applied template. A porous volume is created as a result of the later removal of the template that was utilized as a sacrifice material. As a result, the characteristics of the template used will have a direct impact on the structure of the porous carbon that results. Additionally, doping of carbons, which is a broad term for incorporating any element into the template structure chemically (through grafting) or physically (by insertion/intercalation), provides them distinct activity characteristics due to their capacity to donate or remove electrons, confers on them. Overall, the template synthesis is an original strategy for creating porous carbons, which are more difficult to make using standard methods [50]. New research techniques for creating template-assisted carbons have been developed during the past 10 years, and they have found use in a variety of fields. The majority of these techniques involve an inverse copy of the precursor materials that are utilized as templates to produce carbons with the appropriate porous properties, including carbon nanotubes and mesoporous, macroporous, and microporous carbons [51]. Figure 3 illustrates the methods for the synthesis of porous carbon templates.

Template-based synthesis is a versatile method for creating porous carbon nanofibers (CNFs) with precise control over pore size and distribution. It utilizes templates such as electrospun polymer fibers or sacrificial materials like silica or metal oxide particles. This approach is advantageous for producing ordered porous structures and introducing doping elements, enhancing CNFs’ functionality. However, it involves a complex multistep process, including template deposition, carbonization, and template removal, which can increase production costs and complexity. Template-based synthesis holds promise in various fields, including energy storage and catalysis, thanks to its ability to create tailored porous CNFs.

### 2.3. Chemical Vapor Deposition (CVD)

A chemical reaction that takes place on or near a typically heated substrate surface produces a solid substance that is then deposited from a vapor in a process known as chemical vapor deposition (CVD) [52]. The resultant solid substance might be a single crystal, a thin layer, or both. materials with a wide variety of physical, tribological, and chemical characteristics may be developed by changing experimental circumstances, including substrate material, substrate temperature, composition of the reaction gas mixture, total pressure gas flows, etc. [53]. CVD is another widely employed technique for synthesizing porous carbon nanofibers. In this method, a carbon-containing precursor gas, such as acetylene or ethylene, is introduced into a reactor along with a catalyst. The precursor decomposes on the catalyst surface, leading to the formation of carbon nanofibers. By adjusting the reaction conditions, such as temperature and gas flow rates, the pore structure of the resulting nanofibers can be tailored. Chemical vapor deposition (CVD) and other methods have all been used in research to examine different CNF products [54,55,56]. CVD is seen as a viable approach for producing CNFs. Due to its benefits over other techniques, including its accessibility to a wide range of raw materials, the moderate reaction conditions it experiences, the ease of its installation, the control it has over heat and mass transfer, and its appropriateness for large-scale continuous production [57]. The three basic components are temperature, catalyst, and carbon supply. Methane cracking is an endothermic process; therefore, raising the temperature will enhance the pace at which methane is converted, but doing so will also cause a significant buildup of carbon at the catalyst’s active sites, hastening the catalyst’s deactivation. In general, the 500–1500 °C temperature range is used to prepare CNFs by CVD. CNFs possess inherent porosity, divided into microporosity and mesoporosity, both crucial factors in their characteristics. Microporosity in CNFs comprises tiny pores on the nanoscale, usually less than 2 nanometers in diameter, formed due to disordered carbon atom arrangements and structural defects. These micropores grant CNFs a large surface area, rendering them ideal for gas adsorption and catalytic applications [58,59]. The yield and shape of carbon that is deposited are significantly influenced by temperature, which is a function of reaction rate. Zhou and their team characterized CNF morphology produced through bias-enhanced microwave plasma-enhanced chemical vapor deposition (CVD), revealing a unique wedge-shaped carbon film structure. Their study unveiled the intricate three-dimensional structure of CNFs and the growth process involving carbon gas molecule decomposition, carbon atom diffusion through Ni particles, and deposition at the fiber–particle interface [60].

**Figure 4:**
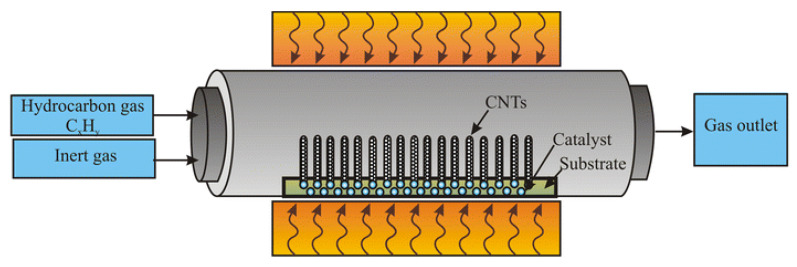
Schematic diagram of a simplified chemical vapor deposition (CVD) system. Adapted from Ref. [69], originally published under a CC-BY license.

The fabrication of CNFs has drawn the attention of several researchers, particularly those who employ the electrospinning method and the chemical vapor deposition (CVD) approach [61,62]. Due to its great controllability, high yield, and simplicity in scaling up, this process has been widely employed for the industrial manufacture of CNFs [63,64,65]. Adsorbents [66], power generation [67], electrodes, and other disciplines [68] are among the many applications for carbon nanofibers created by CVD in the interim. One of the best processes for creating CNFs is CVD, which has been used in a variety of industries [69]. Figure 4 presents a schematic diagram of a simplified chemical vapor deposition (CVD) system.

Chemical vapor deposition (CVD) is a versatile method for producing customized porous carbon nanofibers (CNFs). It allows precise control over factors like temperature, gas flow rates, and catalyst selection to tailor CNF properties. CVD offers accessibility to diverse raw materials and is suitable for large-scale production. However, temperature control is crucial to prevent carbon buildup and catalyst deactivation. Despite its complexity, CVD is favored for CNF production, with applications spanning adsorbents, power generation, and electrodes.

### 2.4. Activation Processes

**Figure 5:**
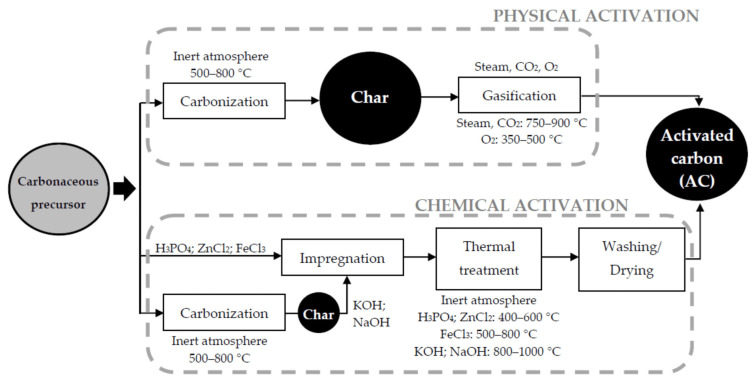
Schematic representation of the step-by-step process for the production of activated carbon. Reprinted from Ref. [70], originally published under a CC-BY license.

Porosity in carbon nanofibers can be enhanced through activation processes. Figure 5 shows the several steps of the activated carbon production.

This involves subjecting the carbon nanofibers to a high-temperature treatment in the presence of activating agents, such as steam, or chemicals like potassium hydroxide or phosphoric acid. Activation leads to the development of additional pores or the enlargement of existing ones, resulting in increased surface area and improved adsorption properties. Furthermore, a binder must also be added during the preparation process, which raises resistance and dead weight and reduces the performance of the supercapacitors [70].

CNF materials attract the attention of researchers and technologists owing to their dimensions and properties. They can function as electrodes for supercapacitors and lithium-ion power cells, supports for catalysts and electrocatalysts, and as parts of composites and chemical sensors [71,72]. In many instances, the specific surface area (Ssp) of such materials—which is quite low in the most commonly accessible materials determines how well they may be used. There are numerous ways to increase (activate) the Ssp value of carbon compounds, including partial oxidation with air, water vapor, CO_2_, molten alkalis, and other reagents. Alkalis are the most often utilized among them to create the surface and porosity [73]. It has been demonstrated that chemical activation is a highly effective technique for obtaining carbons with high surface area and narrow micropore dispersion. The sample is activated at a lower temperature with chemical activation than with physical activation, which is the main benefit. Other benefits include obtaining larger yields, more porosity, and activating the sample in a shorter amount of time. The requirement for a thorough cleaning phase owing to the inclusion of impurities resulting from the activating agent, which may influence the chemical characteristics of the activated carbon [74], and the chemical activation process’ corrosiveness [75] are two major drawbacks. The carbon nanofibers, on the other hand, exhibit a nanostructure made of stacked graphite layers, which is more suited for activation and adsorption processes [76]. Investigating the impact of various activation settings on the final porous texture of the material is required in order to optimize the activation process and subsequently enhance the performance of the activated carbon material. The activation process is greatly influenced by a number of variables, including the raw material, the KOH/carbon material ratio, the activation temperature, and the activation duration. Numerous studies have been conducted on these variables [77]. Sasono et al. prepared activated carbon nanofibers (ACNFs) using electrospinning techniques with coconut shell charcoal as the primary starting material. These ACNFs exhibited an impressive specific capacitance of 186.50 F/g. The resulting ACNFs show significant potential as a low-cost, renewable material with high capacitance [78]. Yang et al. produced carbonized and physically activated polyacrylonitrile (PAN)-based nanofibers. PANI formed tendril-like structures, which improved the electrochemical performance. PANI-deposited ACNF exhibited higher current areas and a specific capacitance of 832 F/g (vs. 353 F/g for ACNF) at 1 A/g in galvanostatic charge/discharge. After 1000 cycles at 1 A/g, PANI-ACNF retained 92% of its specific capacitance (765 F/g). PANI nanostructures facilitated rapid doping/dedoping with electrolyte ions compared to ACNF electrodes [79]. A lesser amount of research has been conducted on other characteristics, such as the composition of the activation agent and the type and flow of the protector gas, but they are nonetheless crucial [80]. 

Activation processes offer several advantages for porous carbon nanofibers (CNFs). They significantly enhance porosity and surface area, making CNFs suitable for various applications such as electrodes and catalyst supports. Chemical activation, in particular, enables low-temperature processing, shorter activation times, and higher yields compared to physical activation methods. However, there are notable disadvantages to consider. Chemical activation may introduce impurities from activating agents, potentially affecting the chemical properties of the resulting CNFs. Additionally, the process can be corrosive. 

### 2.5. Other Methods

There are additional techniques for producing porous carbon nanofibers, such as hydrothermal carbonization, where carbonization occurs under high-pressure and high-temperature aqueous conditions. In addition, physical mixing of carbon precursors with porogens or nanoparticles can be used to introduce porosity. These methods offer alternative routes to achieving specific pore architectures and functionalities. One of the most promising conversion processes is hydrothermal carbonization [81]. Wet pyrolysis, also known as hydrothermal carbonization (HTC), has attracted more attention in recent years because of how straightforward the process is. This includes the fact that biomass does not need to be dried before treatment, which saves energy by skipping the drying process [82]. In actuality, the hydrothermal reaction media includes the water found in natural precursors. In order to distinguish it from biochar produced by pyrolysis, the solid by-product of HTC is given the term hydrochar. HTC provides a number of benefits over dry pyrolysis, including lower operating temperatures, less ash generation, and a greater concentration of functional groups containing oxygen in the solid output [83]. Carbon nanofibers have the inherent benefit of precisely regulated structures. Furthermore, some molecules can have high charge-storage capacity or electrochemical activity for a particular application by doping carried out via elemental physical insertion into the structure or chemical grafting. They have different applications for templated porous carbon, such as adsorption, catalysis, electrochemical energy conversion (lithium-ion batteries, supercapacitors, fuel cells), materials processing, and other applications [84]. Tradler et al. investigated the feasibility of hydrothermal carbonization for food waste conversion. Food waste from restaurants was processed at 200 °C for 6 h. The hydrocarbon obtained has similar fuel properties to lignite. Ultraviolet radiation treatment reduced the process water emission to a minimum [85]. Veltri and colleagues outlined a sustainable approach to producing porous carbon materials through hydrothermal carbonization (HTC). This process involves the decomposition of biomass precursors, producing solid hydrochars in addition to liquid and gaseous byproducts. The resulting porous microspheres exhibit partial graphitization, high nitrogen content, a specific surface area of 1725 m^2^/g, and a pore size distribution that makes them highly suitable for use as supercapacitor electrodes [86].

## 3. Stabilization and Carbonization Process of Nanofibers

Stabilization and carbonization are two crucial steps in the production of carbon nanofibers from precursor materials such as PAN or pitch by electrospinning or other techniques. These steps are crucial for converting the precursor fibers into carbon nanofibers with desired properties. After the electrospinning process, the precursor fibers undergo stabilization treatment. During stabilization, the fibers are heated to temperatures between 200 °C and 300 °C in an oxidative atmosphere, usually air. During stabilization, several reactions take place in the precursor fibers, e.g., cyclization, hydrogenation, etc. During cyclization, the precursor polymer undergoes cyclization reactions and forms a ladder-like or cross-linked structure. In the case of PAN-based nanofibers, this process involves the formation of cyclic nitrile groups [87]. On the other hand, stabilization during dehydrogenation also involves the removal of hydrogen atoms from the polymer backbone, leading to the formation of conjugated double bonds. This step contributes to the formation of a sp^2^ carbon structure. The stabilization process strengthens the fibers by giving them a more robust structure and increasing their thermal stability [88]. It also prevents the precursor fibers from melting or losing their shape during the subsequent carbonization process. Stabilization is a critical step involved in transforming the starting polymer. It facilitates thermally stable structure formation, prevents excessive fiber shrinkage, and can introduce heteroatoms for tailored properties [89]. 

After the stabilization step, the stabilized fibers undergo carbonization, in which they are exposed to high temperatures in an inert atmosphere such as nitrogen or argon. Carbonization is usually performed at temperatures between 800 °C and 1500 °C, depending on the desired properties of the carbon nanofibers [90]. During carbonization, the precursor fibers undergo further structural transformations such as dehydration, graphitization, mass loss, etc. In dehydration, the remaining volatile components, such as water and other gases, are removed in the initial stages of carbonization [91]. In graphitization, the carbonization process results in the re-arrangement of carbon atoms, leading to the development of a more ordered graphitic carbon structure. This graphitization process improves the electrical conductivity and mechanical strength of the carbon nanofibers [92]. Carbonization releases volatile byproducts, including carbon monoxide, carbon dioxide, and other gases. This loss of mass reduces the weight and volume of the fibers. The specific parameters of the carbonization process, such as temperature, heating rate, and duration, can significantly affect the final properties of carbon nanofibers. Carbonization refines nanofibers by enhancing graphitization, crystallinity, surface area, pore structure, carbon content, electrical conductivity, tailored pore shapes, chemical stability, and mechanical properties [93]. These characteristics are vital for energy-related applications. Variations in carbonization parameters, including temperature, heating rate, and duration, allow tailored properties. Higher temperatures eliminate non-carbon elements and volatiles, enhancing graphitic content and strength, suitable for supercapacitor electrodes. Lower temperatures preserve some impurities, potentially increasing surface area, which is valuable for adsorption or catalysis. Heating rate influences purity, while longer durations improve graphitization, resulting in high-purity CNFs with versatile properties for various applications in science and industry [94,95,96].

The advantages of the stabilization and carbonization processes in producing carbon nanofibers (CNFs) include structural strengthening and customizable properties. However, these processes may incur higher production costs and have potential environmental impacts, depending on the energy sources used.

## 4. Energy Storage and Power Generation Applications

### 4.1. Dye-Sensitized Solar Cells (DSSCs)

**Figure 6:**
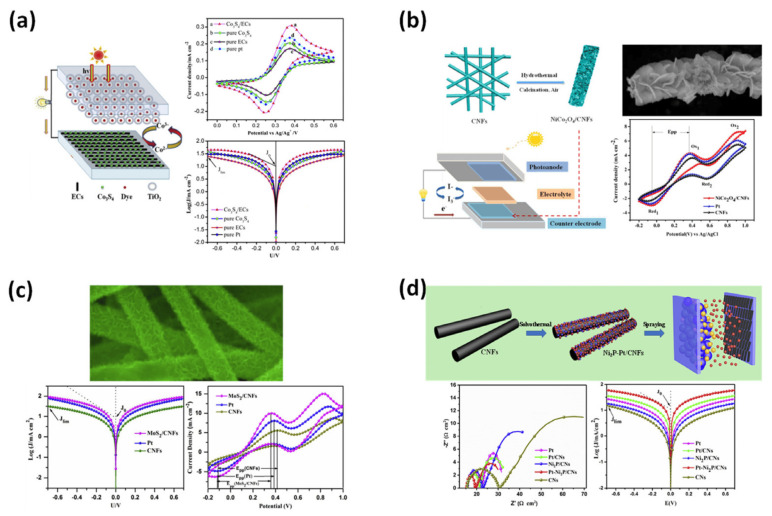
(**a**) CNFs decorated with Co_2_S_4_ and the performance in DSSC device; (**b**) flower-petal−like SiCo_2_O_4_ grown on CNFs used as counter electrode for DSS; (**c**) CNFs grow MoS_2_ mini−sized petals for solar systems; (**d**) CNFs decorated with Ni_2_P and Pt nanoparticles for use as the counter electrode in DSSCs. Reprinted from Ref. [99], originally published under a CC-BY license.

Incorporating electrospun porous carbon nanofibers into DSSCs can improve the efficiency, light absorption, charge transport, and overall performance of these solar cells. Their unique properties make them promising candidates for improving the performance of DSSCs and advancing the field of renewable energy technologies [97]. Here are some specific applications of electrospun carbon nanofibers in DSSCs, such as counter electrodes, conducting frameworks, dye absorption enhancement, light scattering layers, charge collection networks, electron transport media, etc. Electrospun carbon nanofibers are ideally suited as counter electrodes due to their high electrical conductivity and large surface area. The cross-linked nanofiber structure provides efficient charge transfer and enhances catalytic activity [98]. Figure 6 shows the CNFs decorated with Co_2_S_4_ and their performance in a (DSSC). The counter electrode for DSSCs with SiCo_2_O_4_ structures resembles petals grown on CNFs. CNFs promote the growth of petal-like miniature MoS_2_ structures for use in solar systems. CNFs with Ni_2_P and Pt nanoparticles can be used as counter electrodes in (DSSCs).

The nanofiber network provides a three-dimensional structure that enhances dye absorption and electron transport, leading to higher device performance [99]. Carbon nanofibers can be functionalized with dyes or dye sensitizers to improve light absorption in DSSCs. The large surface area of electrospun carbon nanofibers enables effective immobilization of dye molecules, resulting in increased light collection efficiency for the device [100]. The high electrical conductivity of the carbon nanofibers facilitates efficient electron transport from the photoanode to the counter electrode, minimizing electron recombination losses and improving the overall efficiency of the device [101]. The random arrangement of the nanofibers helps to scatter and trap light, increasing the optical path length and improving light absorption in the photoactive layer of the solar cell. This network helps in the rapid collection and transport of photogenerated electrons, minimizing charge recombination and improving the efficiency of the device [102]. Sun et al. have fabricated MoS_2_ carbon nanofiber composites using the electrospinning technique from ammonium thiomolybdate (VI) and polyvinylpyrrolidone. They have shown that the carbon nanocomposites improve the DSSCefficiency by 5.7% when used as counter electrodes [103]. Joschi et al. have investigated electrospun carbon nanofibers as counter electrodes, finding that they have low charge-transfer resistance, large capacitance, and fast reaction rates for triiodide reduction as electrocatalysts and can be provided a cost-effective alternative to platinum (Pt) for triiodide reduction in (DSSCs), which was investigated by using electrochemical impedance spectroscopy and cyclic voltammetry [104]. López-Covarrubias et al. described that the use of electrospinning technology in combination with the use of metal compounds could provide a great approach for the development of DSSCs with superior efficiency and high stability and durability [105]. Zhao et al. successfully synthesized and investigated carbon nanofibers supported by Pt and Ni_2_P nanoparticles as counter electrodes for DSSCs for the first time. DSSCs using Pt-Ni_2_P carbon nanofibers as counter electrodes show excellent photovoltaic performance (efficiency of 9.11%), which is much higher than the conventional Pt carbon nanofiber counter electrode (efficiency of 8.35%) due to the collective effect of high electrical conductivity of carbon nanofibers [106]. Aboagye et al. electrospun polyacrylonitrile (PAN) nanofibers and subsequently demonstrated controllable growth of Pt nanoparticles on the surface of the obtained carbon nanofibers by redox reaction. The hierarchical carbon nanofibers with Pt nanoparticles on the surface were then used as a low-cost counter electrode in DSSCs by stabilizing and carbonizing the carbon nanofibers with Pt nanoparticles on the surface. Compared with the conventional counter electrode, the counter electrode fabricated from carbon nanocomposites exhibited a higher open circuit voltage [107].

While electrospun carbon nanofibers hold promise as counter electrodes in DSSCs due to their low cost, versatility, and sustainability, addressing challenges related to electrical conductivity, catalytic activity, durability, material purity, scalability, cost, and compatibility with tandem devices is crucial for their widespread adoption in commercial DSSC applications.

### 4.2. Fuel Cell Membrane Electrodes

**Figure 7:**
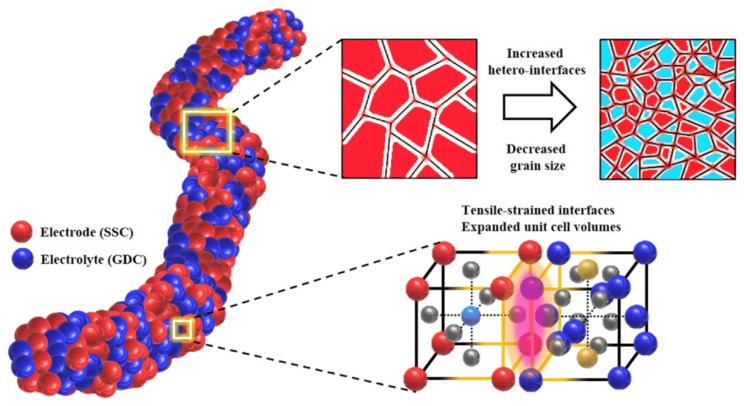
Schematic of electrode/electrolyte nanofiber from Sm_0.5_Sr_0.5_CoO_3−δ_ (SSC) and Gd_0.2_Ce_0.8_O_1.9_ (GDC) with increased hetero-interfaces, decreased grain size, and expanded unit cell volumes. Reprinted from Ref. [109], originally published under a CC-BY license.

Electrospun porous carbon nanofibers can improve the electrode performance of fuel cell membrane electrodes, increase reactant transport, and optimize the overall efficiency of fuel cell systems. These advances contribute to the development of more efficient and sustainable energy conversion technologies [108]. In addition, electrospun porous carbon nanofibers have been shown to have significant potential for membrane electrode applications, including proton-exchange membrane fuel cells and gas diffusion electrodes in fuel cells [109]. Figure 7 shows a schematic representation of Sm_0.5_Sr_0.5_CoO_3−δ_ (SSC) and Gd_0.2_Ce_0.8_O_1.9_ (GDC) electrode/electrolyte nanofibers with increased hetero-interfaces, decreased grain size, and expanded unit cell volume.

On the one hand, the interconnected porous structure of nanofibers enables efficient gas diffusion and transport and provides uniform distribution of reactants and products within the fuel cell [110]. Electrospun carbon nanofibers can be integrated into the electrodes of proton-exchange membrane fuel cells (PEMFCs). The porous nature of the nanofibers facilitates proton transport, which ensures efficient ion exchange through the fuel cell membrane and promotes high-performance fuel cell operation. Waldrop et al. investigated electrospun materials that are of great interest to the energy sector, especially for proton-exchange membrane fuel cells, due to their tunability and durability. Conventional PEMFC electrodes produced by methods such as spraying or coating are unsuitable for large-scale use. Electrospun fiber materials are a promising alternative because they carry catalysts and serve directly as electrodes. This approach improves PEMFC durability and performance with lower catalyst loading, which is critical for the commercialization of PEMFC electric vehicles [111]. Delikaya et al. obtained porous carbon felt structures by phase separation in the shell followed by carbonization treatment and investigated in fuel cell tests. Full cell tests (0.6 mg Pt cm^−2^) show a 21% increase in power density normalized to platinum content compared to the spray-coated reference (1 mg Pt cm^−2^) [112]. Iskandarani et al. developed new materials and strategies to improve the performance of PEM fuel cells at low humidity, and platinum loading is becoming increasingly important. In this study, the fabrication of electrospun sulfonated silica (S-SiO_2_) as a flexible, free-standing, and highly effective novel cathode structure based on poly(vinylidene fluoride-co-trifluoroethylene) for PEM fuel cells was presented [113]. Yusoff and Shaari fabricated nanofibers using electrospinning technology and provided an overview of the electrospinning process by explaining the operating principle and parameters that specifically affect the fabrication of electrospun nanofiber membranes and fiber materials as electrochemical catalysts in fuel cell applications [114]. The ethanolamine (MEA)s with Nafion in salt form (Na+, Li+, or Cs+ as sulfonic acid counterion) were prepared by Waldrop et al. They showed little or no change in maximum power density when the relative humidity (RH) of the feed gas was lowered from 100% to 40%, while the eMEAwith H+-Nafion/peroxyacetic acid (PAA) fibers showed a 33% power loss at 40% RH. The higher power densities at low humidity were attributed to capillary condensation of water in porous fibers with a pore diameter of 1.25 nm or less [115].

### 4.3. Catalysts

The unique properties of electrospun porous carbon nanofibers, such as their large surface area, excellent electrical conductivity, and tunable porosity, make them attractive candidates for catalytic applications [116]. For example, electrospun porous carbon nanofibers can be used as catalysts in various electrochemical reactions such as oxygen reduction reactions (ORRs), hydrogen evolution reactions (HERs), and oxygen evolution reactions (OERs) [117]. The high electrical conductivity and large surface area of nanofibers facilitate efficient charge transfer and enhance catalytic activity. Carbon nanofibers can be functionalized with metal nanoparticles such as platinum, palladium, or other transition metals to enhance the catalytic activity for fuel oxidation and oxygen reduction reactions and create heterogeneous catalysts for various chemical reactions that provide a high number of active sites for catalytic reactions, resulting in enhanced reaction rates and selectivity leading to improved fuel cell performance [118]. Functionalized nanofibers can promote the activation of persulfate or other oxidants, leading to the efficient degradation of pollutants in water or air [119]. As catalysts in energy conversion and storage devices, such as supercapacitors or lithium-ion batteries, porous carbon nanofibers can improve electrochemical performance by improving charge transfer kinetics and increasing electroactive surface area [120]. Also, as catalysts in various chemical synthesis reactions, including organic transformations, hydrogenations, oxidations, and coupling reactions, the large surface area and catalytic activity of nanofibers facilitate efficient reaction pathways and promote the formation of the desired product in chemical industries [121]. Due to their unique properties, electrospun porous carbon nanofibers are attractive candidates for a variety of catalytic processes, offering potential advances in areas of energy generation and storage applications [122]. Guerrero-Pérez et al. discussed the use of carbon nanofibers in catalysis because catalytic support is needed for electrocatalytic applications required for the development of fuel cells, water treatment systems, and batteries [123]. In the Liu et al. study, base carbon catalysts are investigated as the most promising alternatives to modern Pt/C catalysts for the oxygen reduction reaction. The Fe_2_C/CNF catalyst shows uniform dispersion and narrow size distribution of Fe_2_C nanoparticles embedded in CNFs. The obtained catalyst exhibits a positive onset potential (0.87 V vs. RHE) and a large kinetic current density (1.9 mA cm^−2^) and almost follows the effective four-electron pathway, suggesting excellent electrocatalytic activity for ORRs in 0.1 M KOH solution. Moreover, its stability is better than that of the commercial Pt/C catalyst, which can be attributed to the strong binding force between Fe_2_C particles and CNFs. This strategy opens new avenues for the design and efficient production of promising electrocatalysts for ORRs [124]. Woo et al. integrated the widely used Ni_2_Fe catalyst with high oxygen evolution activity (OER) into carbon nanofibers with Ni_2_Fe. To evaluate their overall electrochemical properties for water splitting, Co-CeO_2_ carbon nanofibers and Ni_2_Fe carbon nanofibers were used as HER and OER electrocatalysts in an alkaline electrolyzer. Due to the conformal incorporation of the nanoparticles into the carbon nanofiber, the electrocatalysts exhibit considerable long-term stability of over 70 h of total water splitting [125]. Ponomarev et al. electrospun nanofibers in formic acid with further treatment in the H_2_ stream at 500 °C, which resulted in intense sintering of the platinum particles, forming conglomerates of 50 nm in size, but the individual particles were still less than 10 nm in size. The electrochemically active surface area of the Pt/C catalyst was measured by electrochemical hydrogen adsorption/desorption measurements in 0.5 M H_2_SO_4_ [126].

### 4.4. Sensors Applications

**Figure 8:**
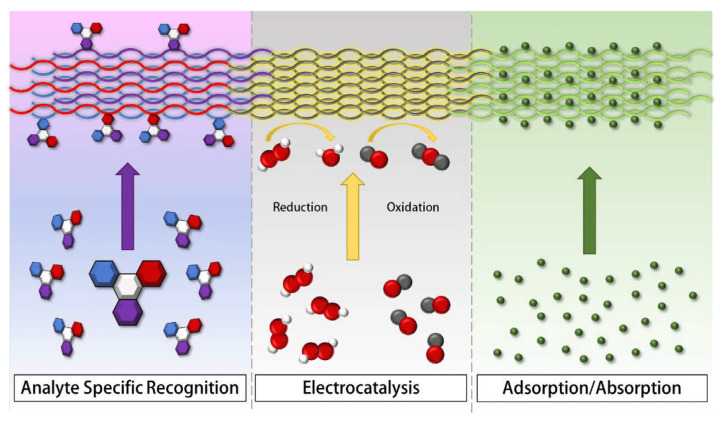
Chart depicting the main detection mechanisms employed by nanofiber-based sensors. On the left: analyte-specific recognition (ASR)—the fibers’ colors correspond to the functional group color of the analyte they adhere to, highlighting specificity. In the center: electrocatalysis (EC)—nanofibers facilitate electron flow, catalyzing reduction or oxidation reactions. On the right: adsorption (AD)—molecules are captured on the nanofiber surfaces, facilitated by their high porosity and extensive specific surface area. Reprinted from Ref. [128], originally published under a CC-BY license.

The sensor applications of electrospun carbon nanofibers are diverse. Their unique properties make them versatile and suitable for various sensing applications in fields such as environmental monitoring, healthcare, industrial sensing, and many others. Electrospun carbon nanofibers have various sensor applications due to their unique properties, such as high surface area, excellent electrical conductivity, mechanical strength, and chemical stability [127]. Figure 8 depicts the primary sensing mechanisms employed by nanofiber-based sensors. It showcases analyte-specific recognition (ASR), where fiber colors match the functional group color of the bound analyte, highlighting specificity. In the center is electrocatalysis (EC), where nanofibers facilitate electron flow for catalyzing reduction or oxidation reactions. To the right, adsorption (AD) is illustrated, where molecules adhere to nanofiber surfaces due to their high porosity and extensive specific surface area.

### 4.5. Biosensors

Electrospun carbon nanofibers can be used as biosensors due to their high sensitivity, fast response, and biocompatibility, making them promising candidates for various biomedical and diagnostic applications [129]. Electrospun carbon nanofibers can be functionalized with specific biomolecules such as enzymes or antibodies, glucose, DNA, proteins, or other biomarkers by measuring changes in electrical conductivity or other electrical properties to create biosensors [130]. These biosensors can detect biological analytes. For example, electrospun carbon nanofibers can be functionalized with the enzyme glucose oxidase to produce glucose sensors. Sensors based on electrospun carbon nanofibers can detect and quantify glucose levels in biological samples such as blood or saliva by measuring the changes in electrical conductivity that result from the enzymatic reaction [131]. In addition, electrospun carbon nanofibers functionalized with single-stranded DNA probes can be used as DNA sensors. The complementary DNA strands in the sample hybridize with the immobilized DNA probes on the nanofibers, resulting in changes in electrical properties that can be measured. This approach enables the detection and identification of specific DNA sequences [132]. Moreover, electrospun carbon nanofibers can be functionalized with specific antibodies or proteins to produce protein sensors. The antibodies immobilized on the nanofibers selectively bind to the target protein analytes, resulting in changes in electrical conductivity that can be measured to detect and quantify proteins [133]. In the same way, electrospun carbon nanofibers can be modified with various enzymes to produce enzymatic biosensors. The catalytic activity of the enzymes on the nanofiber surface triggers specific chemical reactions that lead to measurable changes in electrical properties. Enzymatic biosensors can be used for the detection of various analytes, including metabolites, toxins, or environmental pollutants [134]. Hu et al. have described a polarized poly(vinylidene fluoride-trifluoroethylene)/barium titanate nanofiber mat as the sensing layer, a polyimide film with arrays of circular cavities as the substrate, and a poly(methyl methacrylate) column as the cilium. This bioinspired flexible lateral line sensor with hydrodynamic sensing capability shows promising applications in underwater robotics for real-time flow analysis [135]. Sengupta et al. reported a cilia-inspired high aspect ratio titanium column on an electrospun carbon nanofiber sensing membrane. Calibration experiments on flow sensing demonstrate the feasibility of the proposed method to develop low-cost yet sensitive flow sensors that will be useful for applications such as precise flow monitoring in microfluidic devices, accurate monitoring of air/oxygen delivery in hypoxic patients, and other biomedical devices for monitoring intravenous infusions or urine flow [136]. Kim et al. presented electrospun polyvinylidene fluoride–graphene oxide hybrid nanofibers in aligned mode, which were tested for their multiple efficacies as a locomotion detector, bio-e-skin, smart chair, etc., using the proposed system [137]. Devarajet al. presented that the actual M13 bacteriophage-based multilayer biofilm with porous nanostructures with a diameter of about 150–500 nm and a depth of about 15–30 nm agrees well with the experimental and simulation results, showing that the fabricated multilayer biofilms can be used in sensing, filtering, plasmonics, and biomimetics [138]. Zheng et al. presented a new class of strain sensors fabricated from cellulose nanofibers and graphene in a poly(vinyl alcohol)borax hydrogel with promising applications in the field of wearable sensing [139].

### 4.6. Electrochemical and Chemical Sensors

Electrospun carbon nanofibers find extensive applications in the development of electrochemical and chemical sensors. Their unique properties, such as high surface area, excellent electrical conductivity, and chemical stability, make them suitable for various sensing applications [140]. 

Electrospun carbon nanofibers can be used as electrode materials in electrochemical sensors. They provide a large surface area for efficient electrochemical reactions and electron transfer [141]. Due to their specific properties and porous structure, they can be used to develop pH sensors, gas sensors, biosensors, etc. For example, electrospun carbon nanofibers can be used as pH sensors by measuring the changes in electrochemical potential resulting from the fluctuations in proton concentration [142]. Functionalization of the nanofibers with pH-sensitive materials increases their pH-sensing capability. In the same way, electrospun carbon nanofibers can be used as electrode materials for gas sensors. Adsorption of gas molecules on the nanofiber surface changes the electrochemical properties and enables the detection and quantification of certain gases [143].

Electrospun carbon nanofibers can be used for chemical sensing applications by detecting changes in electrical conductivity or other electrical properties when exposed to certain chemicals [144]. Examples include volatile organic compound sensors, heavy metal ions, environmental sensors, etc. [145]. Electrospun carbon nanofibers can detect and measure volatile organic compounds commonly found in environmental pollutants or in industry. Adsorption of volatile organic compounds on the surface of nanofibers changes the conductivity and enables their detection and quantification. In addition, the functionalization of carbon nanofibers with specific ligands or chelating agents enables selective detection and quantification of heavy metal ions. The binding of metal ions to the functionalized nanofiber surface leads to changes in electrical conductivity or other electrical properties [146]. In the same way, electrospun carbon nanofibers can be used for monitoring and detection of various environmental pollutants, such as water or air pollutants. The high sensitivity and selectivity of nanofibers enable the detection and quantification of certain chemical analytes [147]. The applications of electrospun carbon nanofibers offer enhanced value in the development of electrochemical and chemical sensors for a wide range of applications, including environmental monitoring, industrial sensing, and biomedical diagnostics [148]. Rashid et al. fabricated silver nanofibers. These silver nanofibers were used in sensors to measure relative humidity (RH), ammonia (NH_3_), and temperature. The sensor was of the resistive type and exhibited 4.3 kΩ for the relative humidity (RH%) range of 30–90%, 400 kΩ for NH_3_ (40,000 ppm), and 5 MΩ for temperature detection (69 °C). The durability and speed of the sensor were verified by repeat, response, and recovery tests of the sensor in a humidity and gas chamber [149]. Yoo et al. have fabricated mesoporous electrospun nanofibers by electrospinning with TiO_2_, which exhibit the superior chemical and electrical properties of TiO_2_ and are considered suitable materials for various applications, such as photoelectrodes, photocatalysts, and semiconductor gas sensors [150]. Mehrabi et al. have fabricated nanofibers based on tin dioxide and poly(ethylene oxide) by electrospinning and improved them by calcination and gold doping. The electrospun composite nanofibers were investigated with different doping thicknesses and different calcination temperatures to determine the optimal fabrication parameters leading to high sensitivity [151]. Liu et al. fabricated nanofibers based on oriented polypyrrole (PPy)-coated polyacrylonitrile nanofiber yarn using an electrospinning technique. The electrical responses of the gas sensor based on the PPy-PAN nanofiber yarn to ammonia were studied at room temperature, and the response time was less than 1 s. The above excellent sensor characteristics offer good application potential in the field of ammonia sensors [152].

### 4.7. Batteries 

It is imperative to create energy storage devices that can store electrical energy [153]. Due to its effective storage and distribution of electrical energy, secondary batteries have emerged as one of the most well-known and quickly growing energy storage technologies. There are few publications concerning the creation of porous CNFs and their use as anode materials for rechargeable lithium-ion batteries (LIBs), despite the fact that several possible uses have been envisioned [154,155,156]. Carbon nanofibers are the perfect one-dimensional conductive additives for electrodes due to their high electrical conductivity and great mechanical stability [157]. By electrospinning a bicomponent polymer solution, followed by thermal processing in various atmospheres, porous carbon nanofibers were created. When utilized as anodes for rechargeable lithium-ion batteries, these porous carbon nanofibers’ distinctive structure led to outstanding electrochemical performance, including high reversible capacity and strong cycle stability [158]. Lithium-ion batteries are the most popular rechargeable batteries because of their numerous benefits, including high energy density, safety, lengthy lifespan, and environmental friendliness [159,160]. LIBs are crucial power sources for power tools and contemporary electronics, such as computers and cellphones, and are increasingly employed in drones, electric vehicles, and hybrid electric vehicles [161,162,163,164,165]. The specific energy and power of LIBs, which are essentially constrained by the voltage and cathode capacity, are two of their most crucial properties. The majority of batteries on the market today use complex metal oxides or LiCoO_2_ cathodes [166], which have a variety of drawbacks, including safety concerns. Therefore, finding novel materials with great dependability is very crucial. Zhang et al. extensively investigated the use of MnO_2_ as a cathode material for zinc ion batteries, emphasizing its exceptional electrochemical capabilities. They also investigated the promise of the plasma-induced method for fabricating advanced metal oxide electrode materials optimized for high-performance aqueous zinc ion batteries. In addition, the study addressed the intricacies of plasma-induced ε-MnO_2_ in the field of aqueous zinc ion batteries and shed light on the mechanisms underlying the dissolution and deposition processes of this material [167]. Han et al. explained the process of integrating zinc into nanostructured δ-MnO_2_ in the context of non-aqueous rechargeable zinc metal batteries [168].

### 4.8. Supercapacitors

A supercapacitor is an electrochemical energy storage device with high power capabilities [169], quick charge propagation and charge–discharge operations (in seconds), extended cycle life (greater than 100,000 cycles), low maintenance requirements, and little self-discharging [170,171]. Supercapacitors show potential for various applications due to their advantages of being environmentally friendly, high safety, and ability to operate in a wide temperature range with a nearly infinitely long cycling life. Potential applications include portable electronics (cell phones), memory backup systems, and hybrid cars, where incredibly fast charging is a useful feature [172]. They can be used as load-levelers (backup power for memory, microcomputers, clocks, system boards, etc.) and uninterruptible power supplies (backup supplies intended to defend against power disruption). Due to the mentioned advantages of carbon nanofibers, they are receiving more and more interest as electrode materials for use in supercapacitors [173].

## 5. Applications for Bio-Based Carbon Nanofibers

Bio-based precursors play a decisive role in the production of carbon nanofibers for several reasons. They are more sustainable and environmentally friendly because they can be derived from renewable sources such as plants or biomass, unlike traditional precursors, which are often based on petroleum [174]. This reduces dependence on fossil fuels and reduces the environmental footprint of production. Table 1 provides an overview of the energy-related applications of bio-based carbon nanofibers and shows their multiple benefits in the energy sector. These innovative materials are highly valued for their sustainable and environmentally friendly properties and are therefore an important part of various energy applications such as energy storage, fuel cells, solar cells, and hydrogen storage [175,176,177].

## 6. Challenges and Future Prospects of Research

The electrospun porous carbon nanofiber mats and electrospinning techniques for energy applications represent a promising way to achieve the goals of environmental protection and sustainable energy for future generations. The use of these advanced materials, electrospun porous carbon nanofiber mats, holds significant potential to improve energy storage, conversion, and efficiency, making an important contribution to the transition to cleaner and more sustainable energy systems. Electrospinning is a highly diverse technique with advantages such as adaptability and large surface area but also with limitations in terms of assembly complexity, production speed, solvent consumption, and uniformity. Researchers continue to address these challenges, making electrospinning an area of active development and innovation in nanomaterials manufacturing. In the coming years, it is imperative that further research in this field give high priority to optimizing the methods for producing electrospun porous carbon nanofiber mats. This means focusing on research into innovative methods that not only improve performance but also minimize environmental impact and resource consumption. In addition, concerted action is needed to gain a comprehensive understanding of all potential negative impacts associated with the manufacture and use of these nanofibers to ensure that their use is compatible with sustainability goals. Collaboration between academia, industry, and policymakers will play a critical role in shaping a future in which electrospun porous carbon nanofiber mats play a central role in the pursuit of environmental and energy sustainability.

## 7. Conclusions

This review provides a brief overview of recent advances in the development of electrospun carbon nanofiber mats for energy applications. The purpose of this review is to provide valuable insights that may prove useful to other researchers conducting similar investigations. Electrospun nanofiber materials have become a key component in the field of renewable energy generation and storage, mainly due to their unique properties, such as a high surface-to-volume ratio, a complex pore architecture, and simple manufacturing and processing methods. The porous nature combined with the intrinsic conductivity gives the complex electrospun nanofiber structures the ability to increase the specific surface area of various components such as catalysts, supercapacitors, batteries, sensors, ion-exchange membranes, and electrode materials.

## Figures and Tables

**Table 1:** An overview of the precursors of bio-based carbon nanofibers and their applications.

Precursors	Applications	References
Lignin	Electrodes	[175]
Cellulose, carboxycellulose, plant-based, bio-inspired zwitterionic phosphate groups	Fuel cells	[175,176,177,178]
Protein	Battery anodes and supercapacitors	[179]
Food and bio-based waste	Advanced supercapacitors	[180]
Porous organic polymers	High-performance supercapacitors	[181]
Biomass, chitosan/PEO, chitosan, cellulose, protein	Catalysts	[182,183,184]
Cellulose–nanocarbon composite films	High-performance triboelectric and piezoelectric nanogenerators	[185]
Eggshell	Mechanical energy harvesting	[186]
Lignin, cellulose, and chitin	Energy storage	[187]
Biomass	Supercapacitors	[188]

## Data Availability

Not applicable.
